# Body Temperature, Metabolic, and Circulatory Changes After 8 Days of Water-Only Fasting in Healthy Middle-Aged Men

**DOI:** 10.3390/jcm14165735

**Published:** 2025-08-13

**Authors:** Ilona Pokora, Piotr Wyderka, Wiesław Pilis, Karol Pilis

**Affiliations:** 1 Institute of Sport Sciences, Jerzy Kukuczka Academy of Physical Education, 40-065 Katowice, Poland; p.wyderka@awf.katowice.pl; 2 Collegium Medicum, Jan Dlugosz University, 42-200 Czestochowa, Poland; w.pilis@ujd.edu.pl (W.P.); k.pilis@ujd.edu.pl (K.P.)

**Keywords:** body temperature, fasting, somatic, metabolic and circulatory changes

## Abstract

**Background:** Maintaining thermal homeostasis is a basic function of the human body. This homeostasis depends largely on the body’s nutritional status and other conditions related to it. **Aim:** The present study investigated the impact of 8 days of water-only fasting (8DW-F) on selected features of thermal homeostasis, taking into account somatic, metabolic, and circulatory changes in middle-aged men. **Methods:** A total of 13 healthy men took part in the experiment. Volunteers were examined twice: after a mixed diet (C) and after using 8DW-F. At baseline, the following were recorded: body mass (BM), body fat (FM), fat-free mass (FFM), and total water (TBW), along with basal metabolic rate (BMR) and body surface area (BSA). Then, after 30 min of sitting under thermoneutral conditions, the following measurements were taken: eardrum temperature (Ti), skin temperatures (Tsk), heart rate (HR), systolic blood pressure (SBP), diastolic blood pressure (DBP), oxygen uptake (VO_2_), and respiratory exchange ratio (RER). The following were then calculated: average body (MTB) and skin temperature (MTsk), resting metabolic rate (RMR), body to skin temperature gradient (g), and whole-body thermal conductivity (C). **Results:** The results showed that 8DW-F cause a significant reduction in most somatic variables as well as SBP and BMR (*p* < 0.001), RMR (*p* < 0.05) with no changes in Ti, MTsk, MTB, or C and g *(p* = 0.09). There were also significant correlations between Δ MTB × Δ BMR (*p* < 0.05) and Δ RMR × Δ VO_2_ (*p* < 0.001). Moreover, changes in the C range correlated with Δ RMR (*p* < 0.005) and Δ DBP (*p* < 0.05). **Conclusions:** 8DW-F reduced resting metabolic heat production in the studied men, but sufficient heat conservation ensured that thermal homeostasis was maintained under thermally neutral conditions.

## 1. Introduction

In homeothermic organisms, the maintenance of a stable body temperature (Tb) requires efficient metabolic processes responsible for the production of metabolic heat, as well as the control of this heat flow from deep tissues to the environment.

The minimum energy requirement of a homeotherm in a thermally neutral environment to maintain a stable body temperature is related to the resting metabolic rate (RMR) [1,2,3]. At rest, the metabolic rate is synonymous with the rate of heat production [4]. According to the classic study of Benedict [5], the resting metabolic rate depends on body mass, the anthropometric characteristics of the subject, and the state of metabolic tissue activity. At rest, under thermoneutral conditions and normal dietary conditions, internal body temperature is strongly correlated with VO_2_ oxygen uptake and metabolic heat production [6]. On the other hand, the rate of heat loss is determined by the rate of heat conduction from body tissues to the skin, and the rate of heat transfer from the skin to the environment is a function of the magnitude of existing thermal gradients [7]. When metabolic heat production is balanced by the amount of heat lost from the body, the internal body temperature remains stable.

Changes in the amount of “energy” and nutrient availability in food have been found to affect body mass, internal energy stores, metabolism, and resting body temperature [8,9,10,11,12] and also influence the use of energy substrates and the rate of hormonal and thermoregulatory reactions during various types of exercises [13,14].

Recently, studies have demonstrated the health benefits of fasting and recommended it as a treatment method to improve mental and metabolic health, alleviate many health problems, and increase life expectancy [15,16,17,18,19]. These effects were attributed in part to a reduction in body mass, metabolic rate, and body temperature.

Many earlier studies used fasting as a weight-loss intervention and focused on changes in body weight rather than changes in body composition. It has been noted that fat-free mass is the largest contributor to RMR change [20,21,22]; therefore, a significant reduction in FFM results in a lower resting metabolic rate [23], but it also predisposes to fatness during the re-feeding period. Adipose tissue has a lower thermal conductivity than other tissues and therefore can influence the thermal conductivity of the body and is an important part of the insulating barrier for heat loss [24]. Given the relationship between the physical characteristics of the body and heat transfer, body composition assessments are important considerations for modeling heat loss and temperature regulation.

In the previous study, it has been observed that during periods of reduced food availability, both the energy production metabolic rate (RMR) [25] and body temperature decreased [26,27]. According to Landsberg [27], lower body temperature and malnutrition can be considered as a conserved metabolic strategy towards energy conservation during food shortage.

Two major categories of dietary strategies, namely calorie restriction (CR) and fasting, have recently been extensively studied [18]. Evidence suggests that fasting, during which only water is consumed, results in potentially health-promoting physiological effects [8,16,27,28]. In general it has been noted that fasting decreases heart rate (HR) and blood pressure [27,29]. In addition, there are changes in vascular control and skin blood flow [30], skin temperature [15], thermal conductivity [31], heat production [27,32], and heat transfer from the skin to the environment [33].

It has therefore become interesting to test whether, under conditions such as water-only fasting and potentially reduced body mass and heat production, the body can maintain a stable internal temperature under thermoneutral conditions. It seems that this is possible if there are changes in the thermodynamic properties of the organism [7], and suppression of heat loss can compensate for the reduced heat production during fasting [27,32].

There is still insufficient data on the effects of fasting on body temperature, metabolic rate, and circulatory changes during food limitation in middle-aged men. We hypothesized that circulatory changes, as well as modifications in body thermal conductance, may be an integral part of the physiological response to reduced energy availability and weight loss after an 8-day fast in middle-aged men to maintain stability of internal temperature.

Therefore, the purpose of this study was to determine the effects of 8-day water-only fasting (8DW-F) on resting body temperatures, metabolic rate, body weight and composition, and circulatory indices in healthy middle-aged men, and additionally find out the relationship between these variables.

## 2. Materials and Methods

### 2.1. Ethical Approval

Informed consent was obtained from all participants prior to the onset of the study and the study protocol was approved by Committee for Ethics in Scientific Research of Jan Dlugosz University in Czestochowa (Poland; KE-0/1/2019). Experimental procedures were conducted in accordance with the standards set by the Declaration of Helsinki—Ethical Principles for Medical Research Involving Human Subjects. Qualified medical personnel informed the surveyed men about the possible negative health effects of such practices. The inclusion criteria were as follows: (1) previous experience in undertaking DWF of more than three days; (2) age range of 30–70 years; (3) body weight between 60 and 100 kg; (4) body mass index (BMI) between 20 and 29.9 kg/m^2^; (5) no chronic diseases; (6) systolic blood pressure in the range of 100–140 mmHg and diastolic blood pressure in the range of 60–90 mmHg. The exclusion criteria were as follows: (1) the presence of chronic diseases; (2) smoking cigarettes; (3) the use of medications.

### 2.2. Participants

Thirteen healthy middle-aged men who had previously practiced starvation diet (for 3–42 d) participated in this study. The subjects stated that they had practiced regular physical activity in the form of moderate-intensity yoga. Detailed participant characteristics are outlined in Table 1. The subjects were non-smokers with no history of chronic diseases and had current valid medical examinations and showed no contraindications that would exclude them from the study. The participants declared that they did not take any medications or dietary supplements for at least one month prior to the study and did not use any calorie-restrictive strategies for at least the previous 6 months.

### 2.3. Experimental Designs

**Figure 1:**
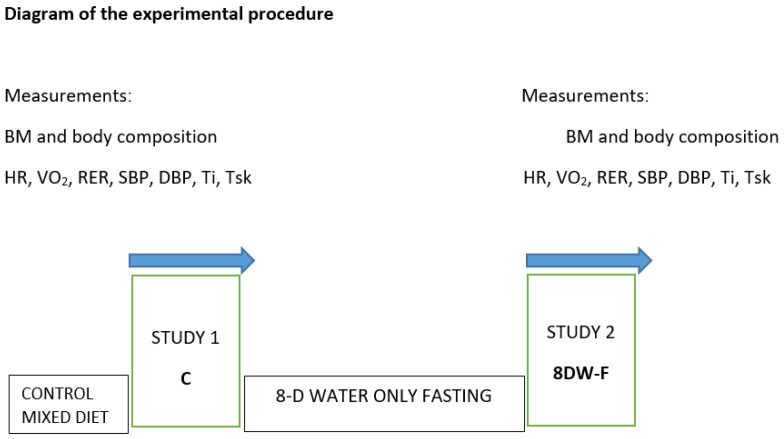
The experimental design of the present study. Legend: BM—body mass; HR—heart rate; VO_2_—oxygen uptake; RER—respiratory exchange ratio; SBP—systolic blood pressure; DBP—diastolic blood pressure; Ti—internal temperature; Tsk—skin temperature; C—study before the fasting intervention; 8DW-F—study after 8-day water-only fasting.

The experimental design of the present study is displayed in Figure 1. The experiment consisted of two phases: a control study before the fasting intervention and a retest after 8 days of water-only fasting (8DW-F).

All participants were examined twice while fasting: after consuming a 3-day mixed diet consisting of 15% protein, 50–65% carbohydrates, and 35–20% fat (examination 1, control—C) and again after 8 days of water-only fasting (8DW-F, examination 2). During the fasting intervention, the volunteers were not permitted to eat anything, but they could drink moderately mineralized water at any time to avoid dehydration. Throughout the eight days of fasting, participants remained in normal, free-living conditions and were encouraged to continue their normal daily activities. Subjects were monitored by a clinician for a period of eight days during the 8DW-F and seven days of refeeding.

### 2.4. Measurements

#### 2.4.1. Body Mass and Composition Measurement

Participants arrived at the laboratory in the morning. At the beginning, age, height, and body mass along with body composition were recorded.

The body height of the examined men was recorded using a stadiometer, Charder HM (Charder HM, Bilcza, Poland).

Body mass and its composition (fat mass—FM; fat-free mass—FFM; total body water—TBW; body mass index—BMI), as well as basal metabolic rate (BMR), were determined using the TANITA BF-300 body composition analyzer (TANITA, Tokyo, Japan). The device utilized a non-invasive bioelectrical impedance analysis (BIA) method. Participants were examined after an overnight fast and after passing residual urine and feces, in accordance with the manufacturer’s instructions.

#### 2.4.2. Circulatory Assessments

Blood pressure—systolic (SBP) and diastolic (DBP)—and heart rate (HR) were recorded at rest after 15 min of sitting. Blood pressure was measured in the left upper arm using a clinically validated automatic blood pressure monitor, Omron 711 Automatic IS (Omron, Matsusaka, Japan), according to the manufacturer’s instructions. Systolic and diastolic blood pressure were the means of two measurements. HR was monitored continuously using a Polar-Electro RS400 telemetric heart rate sensor (Polar-Electro, Kampele, Finland).

#### 2.4.3. Resting Metabolic Rate

After arriving at the laboratory, the volunteers stayed at rest for 30 min during both research sessions at an ambient temperature of 21–24 °C, a relative humidity of 45–55%, and an atmospheric pressure of 750–770 mmHg. Laboratory conditions were determined using the wet-bulb dry temperature (ESI) calculated according to the formula of Moran et al. [34]. Volunteers were shoeless and wearing one layer of clothing during the study. They were also instructed to wear clothing of a similar weight during their next visit to the laboratory.

Resting metabolic rate (RMR) was determined, after an overnight fast, on the last day of the three-day mixed diet (Study 1, Control) and after the last day of the eight-day water-only fasting (Study 2, 8DW-F) by the indirect calorimetry method. In this non-invasive technique, oxygen uptake and respiratory exchange rate (RER) were measured continuously (for 30 min in subjects at rest, in a sitting position in a temperature-controlled room), using a rapid gas analyzer Medisoft, ErgoCard (Medisoft, ErgoCard, Sorinnes, Belgium). Prior to each test, the analyzer was calibrated using a precise gas mixture according to the manufacturer’s instructions.

Only oxygen uptake and respiratory exchange rate (RER) measurements from the last 20 min of measurements were used for calculations of the resting metabolic rate (during which the variables of interest (e.g., VO_2_ L/min), did not differ by more than 5%).

Based on resting VO_2_ and RER, resting metabolic rate (RMR) was calculated according to Nishi’ equation [35] and expressed in watts per unit of body mass (W/kg) and watts per unit of body surface area, (W/m^2^). The RMR value for each patient was taken as the average value obtained during the 20 min recording period.

#### 2.4.4. Body Temperature Measurement

Internal body temperature (Ti) was measured in the ear canal near the eardrum using an insulated molded plug and thermistor Ellab, E-val- Model Flex 1.38 (Ellab, Hillerød, Denmark), isolated from the external environment with cotton wool. Skin temperature (Tsk_s_) was recorded using skin thermistors (Ellab Instruments Temperature Sensors, (Ellab, Hillerød, Denmark) attached with surgical tape to three locations on the left side of the body, i.e., over the pectoralis major muscle (TsCh), over the biceps brachii muscle (TsF), and over the rectus femoris (TsTh). Ti and Tsk_s_ were recorded continuously at 30 s intervals.

### 2.5. Calculations

Body surface area (BSA) was calculated according to the formula of DuBois and DuBois [36]. The mean weighted skin temperature (MTsk) and mean body temperature (MTB) were calculated according to the Burton scale [37] and the Stolwijk and Hardy equation [38]. Mean arterial pressure (MAP) was calculated according to the formula MAP = DBP + (SBP − DBP)/3. The skin-to-environmental temperature gradient was calculated as g = (Ti − MTsk). The whole-body thermal conductivity (C) was calculated according to the equation C = RMR/(Ti − Ta), where Ta is the ambient temperature.

### 2.6. Statistical Analysis

The obtained data is presented as the arithmetic mean (x) and standard deviation (SD). The normality of the data was confirmed using the Shapiro–Wilk test. However, their sphericity was assessed using the Mauchly sphericity test, and in cases of violation of the assumptions regarding sphericity, Greenhouse–Geisser corrections were applied. Taking into account the analyses of the above tests, the significance of differences for individual variables before and after the fasting intervention was assessed using the “t” test for related values, and the percentage difference between Study 1 and 2 was calculated. Standardized mean differences (i.e., Cohen’s d effect size) were calculated as the mean change (C to 8DW-F) divided by pooled standard deviations [39]. Linear regression analysis was used to investigate the association between changes in somatic body components and metabolic rate and body temperature, as well as changes in circulatory variables and body temperature and whole-body thermal conductivity, after 8DW-F. Pearson correlation coefficients were used to identify correlations between the absolute changes in C, somatic variables, metabolic rate, and body temperature. For all statistical analyses, *p* < 0.05 was considered significant. Statistical analyses were performed using STATISTICA 13.0 software (StatSoft, Kraków, Poland).

## 3. Results

### 3.1. The Effect of 8DW-F on Somatic Variables

**Figure 2:**
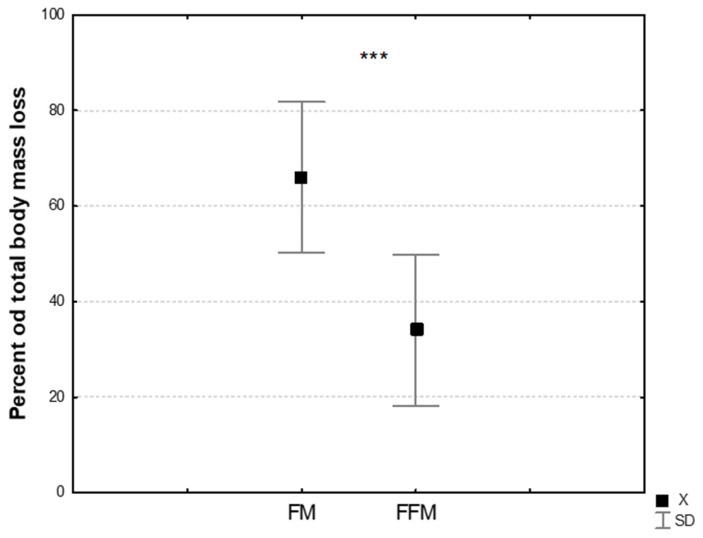
Percentage share of FM and FFM in total body mass loss after 8DW-F. Note: Values are means and SD; FM—fat mass; FFM—fat-free mass; ***—significant difference between FM [%] and FFM [%] mass loss (*p* ≤ 0.005).

The average body mass decreased by 5.42 ± 1.32 kg, and in relative terms by 6.93 ± 1.66% after 8DW-F (Table 1). Body mass loss was attributable to both fat-free mass, 3.76 ± 1.29 kg, and fat mass, 1.98 ± 0.63 kg. Following 8-day fasting (8DW-F), the percentage of fat mass loss was significantly higher compared to fat-free mass loss (Figure 2).

Moreover, the BSA/BM ratio increased by +3.7 ± 0.96% after 8DW-F (Table 1).

### 3.2. Changes in the Circulatory, Metabolic, and Thermal Status Under the Influence of 8DW-F

In terms of changes in the circulatory system after the fasting intervention, only a reduction in SBP (*p* < 0.001) and MAP (*p* < 0.01) was observed (Table 2). Furthermore, following 8DW-F intervention, in terms of metabolism a decrease in BMR was detected (Table 2). The change in basal metabolic rate [%] after 8DW-F was strongly associated with body mass loss [%] and Δ FFM [%] but not FAT [%] loss (Table 3).

**Figure 3:**
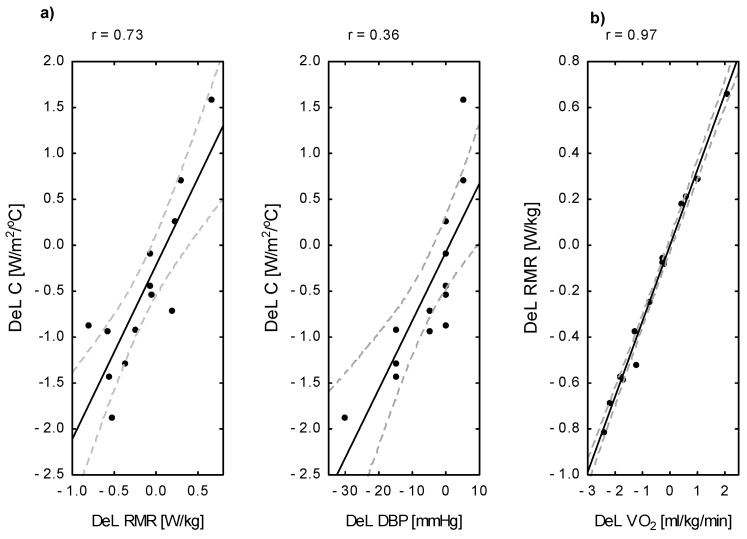
Scatterplots of the relationship between changes (Del) in (**a**) whole-body thermal conductivity and metabolic and circulatory indices, and (**b**) between resting metabolic rate and oxygen uptake after 8 days of water-only fasting. Legend: Del C—change in whole-body thermal conductivity; Del RMR—change in resting metabolic rate; Del VO_2_—change in oxygen uptake; Del DBP—change in diastolic blood pressure after 8DW-F; r— Pearson correlation coefficient.

After an 8-day fasting period (8DW-F), both the absolute oxygen uptake (VO_2_) [L/min] (*p* < 0.05) and the resting metabolic rate (RMR) were lower than before fasting (Table 2). A significant positive correlation was found for Δ RMR × Δ VO_2_ [L/min] (r = 0.97; *p* < 0.001) (Figure 3b). Using regression analysis, only ΔVO_2_ [%] was entered into the model, explaining 98% (R^2^ = 0.97; *p* < 0.001) of the variance in Δ RMR [%].

The internal body temperature of control men was 36.4 °C, and after 8DW-F, Ti reduced by 0.05 ± 0.74 °C. There were no significant differences in MTsk (*p* = 0.068) and MTB (*p* = 0.49) after C and 8DW-F (Table 2). The internal-to-mean skin temperature gradient and whole-body thermal conductivity were lower after 8DW-F with respect to C (*p* > 0.05). The results of the regression analysis showed no significant relationship between the change in internal temperature and Δ RMR [%] and Δ BMR [%] after the 8DW-F intervention. Only Δ g [%] explains 58% (R^2^ = 0.58; *p* < 0.003) of the variance in Δ Ti, and 73% (R^2^ = 0.73; *p* < 0.0002) of the variance in Δ MTsk. The change in MTB [Δ%] after 8DW-F was associated with a BMR decrease [Δ%] (*p* = 0.03) and body mass loss (*p* = 0.01) (Table 3).

There was no association between the change in whole-body thermal conductivity (C) after fasting and Δ FFM (*p* = 0.23), Δ BMI (*p* = 0.31), and Δ BSA. Using regression analysis, only DBP [%] was entered into the model, explaining 56% (R^2^ = 0.56; *p* < 0.01) of the variance in Δ C [%]. The change in whole-body thermal conductivity after 8DW-F was correlated with both the change in resting metabolic rate (r = 0.73; *p* < 0.005) and Δ DBP (r = 0.36; *p* < 0.05) (Figure 3a).

## 4. Discussion

The main finding of the study was that, compared to C intake, 8-day water-only fasting (8DW-F) provided a reduction in body mass, of which 38.2% was FFM and 68% was FM loss. There was also a significant decrease in VO_2_ and resting metabolic rate, suggesting lower metabolic heat production in middle-aged men accustomed to 8DW-F. In addition, 8DW-F reduced whole-body thermal conductivity (*p* = 0.09), and it was found that reduced RMR and DBP best predicted the decrease in C after 8-DWF. However, despite lower heat production and reduced body thermal conductivity after 8 days of water-only fasting, middle-aged men were able to maintain Ti as effectively as in the control group. Thus, 8-day water-only fasting (8DW-F) is safe and may be useful for improving body mass composition and circulatory regulatory function in middle-aged men under thermoneutral conditions, but it would probably not be safe enough to maintain thermal balance in middle-aged men exposed to an environment below the thermoneutral zone.

When considering the production of heat by the human body and its exchange with the environment, attention should be paid to such endogenous factors as the mass and surface area of the body, its composition, and age. Also important are specific tissues heat capacity, thermal conductivity, blood perfusion, metabolic heat production, and water content in the body [40]. Exogenous factors also play an important role in the production and exchange of heat, including the temperature and humidity of the body’s external environment and its nutritional status. The 8DW-F used in our studies resulted in a significant reduction in body weight. Following the reduced body weight, we observed a reduction in basal and resting metabolic rate, which resulted in lower heat production in the studied men. It has been shown that the body’s thermal homeostasis is closely related to its nutritional status [41]. Observations by other authors indicate that the reduction in basal metabolism during fasting is greater than indicated by the amount of body weight reduction [17,42]. In the case of resting metabolism, our research confirms this principle (Δ BM; −6.9 ± 1.7% vs Δ RMR; −14.6 ± 22.9%).

This energy saving during starvation is attributed to “metabolic adaptation” [43,44]. In our studies, this energy saving consisted in retaining endogenous heat in the central (core) part of the body, which ensured the maintenance of balanced systemic thermal homeostasis, the exponent of which was stable Ti. Confirmation of energy saving in fasting conditions in our studies is seen in the significant correlation between changes in BMR and MTB (*p* < 0.05) and the decrease in whole-body thermal conductivity (Δ C; −12.5 ± 27.2%; *p* = 0.09). The linear relationship between the average Δ MTB [%] and the level of restriction of BMR [Δ%] supports the idea that MTB changes are an integral aspect of the effect of energy restriction.

As body mass decreases during fasting, the mass of internal organs decreases [45], including those with a high metabolic rate, such as the brain, heart, liver, spleen, and kidneys [46]. Reducing the mass of these organs during fasting may result in less metabolic heat production in the body [47]. This reduced production of metabolic heat is associated with limited oxygen uptake, expressed in absolute values VO_2_ [L/min] in our studies, and is confirmed by a significant positive correlation of changes in the resting metabolic rate and oxygen uptake in the conditions of fasting intervention (*p* < 0.005).

Reducing the metabolic rate during fasting is also associated with a significant loss and saving of carbohydrates in the body, which influence the rate of fat utilization [48]. Increased fat utilization during long-term fasting is caused by increased activity of the sympathetic nervous system, which leads to the breakdown of adipose tissue triglycerides via the β-3 adrenergic receptor. Therefore, in our studies, a significant reduction in fat tissue mass was observed (−11.2 ± 5.1%). It is suggested that a feedback regulatory loop between adaptive thermogenesis and changes in fat content is activated during fasting [49]. In support of this thesis, there is a significant correlation between reduced thermogenesis (assessed by BMR changes) and fat tissue loss [50,51]. Unfortunately, in our studies, such a relationship was not detected and there was only a tendency towards a positive correlation between the reduction in adipose tissue and the decrease in Tsk in the conditions of fasting intervention (*p* < 0.06).

It should be noted here that the thermal conductivity of adipose tissue is lower than that of other tissues [52], and fat constitutes a thermal insulation barrier protecting the body against heat loss. Reducing fat tissue mass should therefore promote greater thermal conductivity and enhance heat dissipation. However, this has not been confirmed in our research. On the contrary, the whole-body thermal conductivity tended to be reduced (although not statistically decreased) and the Ti–Tsk temperature gradient was reduced under fasting and fat tissue reduction conditions (Δ g −13.2 ± 26.5%; *p* = 0.09).

At the same time, the amount of FFM also decreased significantly (by 5.86%), as well as the above-mentioned oxygen uptake (−10.2%). In order to demonstrate the role of FFM in the body’s thermal status, we calculated the linear correlation coefficient between changes in resting metabolic rate and FFM expressed as a percentage. This coefficient had a positive value but was statistically insignificant (*p* < 0.06). The above-mentioned facts may indicate lower heat production in both adipose tissue and FFM, which was caused by their reduction. However, under 8DW-F conditions, no changes in Ti, MTB, or MTsk were recorded, which clearly indicates that the body saves heat with reduced heat production. Therefore, the expected thermal insulation from adipose tissue did not play a significant role, because under the conditions of its reduction during 8DW-F, the Ti–MTsk gradient did not increase as expected, but decreased (*p* = 0.09). Therefore, it is expected that the lower FFM reduced this temperature gradient in the fasting intervention conditions.

Such autoregulatory changes in body composition during fasting may also influence the body’s thermoregulatory reactions by reducing the water content (−5.7%). This results in a reduction in blood volume and may manifest itself in limited perfusion, mainly of tissues with a high metabolic rate and less heat dissipation from them by this liquid tissue. Unfortunately, we are unable to precisely determine the size of perfusion changes under the influence of the fasting intervention, but it should be assumed that it was reduced because the factors determining it, i.e., mean arterial pressure (MAP) and body mass, were significantly reduced. Analyzing the assumptions of Rossmann and Haemmerich [12], stating that an increase in tissue temperature causes an increase in its perfusion, in our studies the average body temperature did not change, which means that perfusion should not increase, and in heat-saving conditions it should be reduced. Indirect evidence of the functioning of such a mechanism may be a positive linear correlation coefficient calculated in thermoneutral conditions for changes in diastolic blood pressure and thermal conductivity in the tested volunteers (*p* < 0.05).

An important factor in the thermoregulation process is the size of the body surface area through which the body can exchange heat, which in conditions of thermal comfort occurs mainly through radiation [53]. The control group differed significantly in body surface area to body weight ratio from the fasting group, which may have affected their internal temperature. Larger exposed surface area in relation to body weight is a hindrance to temperature regulation and may also impair thermoregulatory control in fasting men. The higher BSA/BM in the 8DW-F group may have allowed more heat to be transferred to the environment. This was not the case, as del BSA and del C were not significantly correlated. In our studies, after 8 days of fasting on water only, body surface area decreased by 3.54%, body weight decreased by 6.81%, and the ratio of body surface area to body mass increased by 4.0%, all of which were significant (*p* < 0.001). Further calculations showed that the positive linear correlation coefficient for changes in thermal conductivity and body surface area occurring under the influence of the fasting intervention did not reach the level of statistical significance (*p* > 0.05). Such somatic changes indicate that a reduction in body surface area decreases, although not significantly, the amount of heat loss from the body. It should be mentioned that peripheral heat dissipation is particularly effective in the limbs, where the surface area to body mass ratio is 4–5-times higher than in the central parts of the body [54].

What is also important for the body’s thermolytic reactions is the demonstration in our research of a statistically significant positive correlation between thermal conductivity and resting metabolism in thermoneutral conditions (*p* < 0.005). This confirms that along with the reduction in resting metabolic rate, thermal conductivity is reduced, i.e., heat is saved.

According to Dulloo and Jacquet [42], adaptive thermogenesis during fasting relies on two distinct systems. The first of them is referred to as non-specific control of thermogenesis, occurring mainly in organs with a high metabolic rate, and is associated with the stimulation of the sympathetic nervous system through factors such as lack of food or weakening of the body’s immunity at that time. The second control system, which operates much slower than the first, is independent of the functional state of the sympathetic nervous system and is called “adipose tissue-specific”. This system only responds to signals resulting from the degree of depletion of fat stores. With regard to predictors of adaptive thermogenesis, reanalysis of BMR and body composition revealed determinants of human variability in the reduced thermogenesis component, which is not related to fat depletion, but probably related to “non-specific” control of thermogenesis. Further detailed research should be carried out to demonstrate the priority importance of any of the above-mentioned systems during famine.

To sum up, it should be stated that in middle-aged men undergoing 8DW-F, somatic, metabolic, and circulatory conditions played an important role in maintaining a thermal balance. The tested volunteers maintained thermal equilibrium described by the constancy of Ti, MTB, and MTsk, despite reduced heat production resulting from reduced body mass and greater fat loss than FFM.

## 5. Limitations and Future Perspectives

The presented study is not free from limitations. Significant factors hindering the analysis and discussion of the results include the relatively small sample size and significant differences in the fasting protocols between our study and the available literature. Moreover, the study was performed only on men, which does not allow us to draw general conclusions regarding in the human population subjected to fasting intervention. Other factors limiting this study may include the age of the study participants, potential technical difficulties in determining body composition, or the use of indirect, less accurate methods in obtaining some results, e.g., MRM or BMR. It should be agreed that an additional measurement of cutaneous blood flow, which was not performed in this study, would describe changes in perfusion in this organ and would indirectly indicate peripheral thermolytic changes. Furthermore, some of the biochemical variables analyzed are subject to natural daily fluctuations, so it would be interesting to assess their levels more than twice, at different times of the day. Another interesting research direction would be to examine the long-term effects of an 8-day fast, for example, a week or a month after the end of the fasting period.

In summary, there is a need for future studies involving larger groups of volunteers, using various fasting protocols (long, short, intermittent) and varying levels of physical activity and female participation. Furthermore, studies should be conducted at multiple time points during fasting, including after a longer period following the completion of the fasting intervention. This would provide a more comprehensive picture of how long-term, water-only fasting affects health and functional capacity.

## 6. Conclusions

This study showed that 8DW-F changed the thermoregulatory mechanisms of the studied men and metabolic heat production decreased during this period. Moreover, it was shown that Ti, MTB, and MTsk did not change and were associated with a decrease in RMR and DBP, suggesting that the mechanisms related to heat loss were effectively suppressed, despite a significant loss of body mass and fat mass and an increase in the body surface area to mass ratio.

## Figures and Tables

**Table 1:** Age and anthropometric data before and after 8DW-F.

Variable	C (*n* = 13) X ± SD	8DW-F (*n* = 13) X ± SD	Δ [8DW-F—C]	Cohen’s d	Δ [%]
Age [years]	51.36 ± 13.0	**----------**	**----------**	**--------**	**--------**
BH [cm]	178.57 ± 4.45	**----------**	**----------**	**--------**	**--------**
BM [kg]	79.62 ± 10.69	74.20 ± 10.77 **	−5.42 ± 1.32	0.52	−6.9 ± 1.7
FM [kg]	15.55 ± 5.66	13.62 ± 5.59 **	−1.98 ± 0.63	0.36	−11.2 ± 5.1
FFM [kg]	64.2 ± 5.65	60.44 ± 5.99 **	−3.76 ± 1.29	0.67	−5.9 ± 2.1
TBW [kg]	47.8 ± 4.13	44.34 ± 4.22 **	−2.67 ± 0.91	0.66	−5.7 ± 1.8
BSA [m^2^]	1.98 ± 0.14	1.91 ± 0.15 **	−0.069 ± 0.02	0.48	−3.5 ± 0.9
BSA/BM [m^2^/kg]	0.025 ± 0.002	0.026 ± 0.002 **	0.0009 ± 0.0003	0.59	+3.7 ± 0.9

Note: Values are means and SD; C—mixed control diet; 8DW-F—8-day water-only fasting; Δ—difference between 8DW-F and control; BH—body high; BM—body mass; FM—fat mass; FFM—fat-free mass; BSA—body surface area; TBW—total body water; BSA/BM—BSA/BM ratio; **—significant difference between C and 8DW-F sessions (*p* ≤ 0.01).

**Table 2:** Metabolic, thermal, and circulatory status of the studied men before and after 8DW-F.

Variable	C (*n* = 13) X ± SD	8DW-F (*n* = 13) X ± SD	Δ [8DW-F—C]	Cohen’s d	Δ [%]
VO_2_ [L/min]	0.39 ± 0.10	0.32 ± 0.12 *	−0.07 ± 0.10	0.60	−16.3 ± 23.2
VO_2_ [mL/kg/min]	4.91 ± 1.13	4.32 ± 1.27	−0.58 ± 1.29	0.50	−10.2 ± 24.2
RER	0.79 ± 0.07	0.83 ± 0.07	0.04 ± 0.09	0.55	5.9 ± 5.2
BMR [kcal]	1706.5 ± 123.9	1648.7 ± 116.9 ***	−75.42 ± 18.6	0.48	−4.4 ± 1.1
RMR [W/kg]	1.64 ± 1.55	1.45 ± 1.56	−0.19 ± 1.67	0.51	−9.9 ± 23.4
RMR [W/m^2^]	65.92 ± 16.43	54.25 ± 19.52 *	−10.0 ± 16.3	0.64	−14.6 ± 22.9
Ti [°C]	36.44 ± 0.52	36.38 ± 0.59	−0.05 ± 0.74	0.46	−0.13 ± 2.04
MTB [°C]	34.69 ± 1.75	34.79 ± 1.86	0.13 ± 0.57	0.17	0.37 ± 1.61
MTsk [°C]	30.57 ± 0.65	31.35 ± 0.60	0.68 ± 1.07	0.78	2.3 ± 3.54
C [W/m^2^/°C]	3.99 ± 1.08	3.53 ± 1.32	−0.47 ± 0.92	0.46	−12.5 ± 27.2
g [°C]	5.82 ± 0.84	4.89 ± 1.0	−0.93 ± 1.51	0.54	−13.2 ± 26.5
SBP [mmHg]	126.42 ± 12.92	112.14 ± 11.38 ***	−14.28 ± 9.77	1.22	−11.01 ± 6.8
DBP [mmHg]	82.5 ± 10.14	75 ± 11.09	−7.5 ± 14.10	0.73	−8.06 ± 11.2
MAP [mmHg]	95.67 ± 10.28	86.14 ± 9.97 **	−9.53 ± 10.91	0.98	−9. 9 ± 10.1

Note: Data are means ± SD; Ti—internal temperature; MTsk—mean skin temperature; MTB—mean body temperature; SBP—systolic blood pressure; DBP—diastolic blood pressure; VO_2_—oxygen uptake; RER—respiratory exchange ratio; BMR—basal metabolic rate; RMR—resting metabolic rate; g—internal-to-mean skin temperature gradient; C—whole-body thermal conductance; MAP—mean arterial blood pressure, rest—resting values; *—significant difference between C and 8DW-F sessions (*p* ≤ 0.05); **—significant difference between C and 8DW-F sessions (*p* ≤ 0.01); ***—significant difference between C and 8DW-F sessions (*p* ≤ 0.005).

**Table 3:** Relationships between relative changes in thermal, metabolic, somatic, and circulatory parameters after 8DW-F in middle-aged men.

Components	Variables	Δ Ti [%]	Δ MTB [%]	Δ C [%]	Δ g [%]	Δ BMR [%]
Temperature	Δ Ti [%] R^2^	—	—	—	0.58 ***	—
	Δ MTsk [%] R^2^	—	—	—	0.73 ***	—
Metabolic	Δ VO_2_ [%] R^2^	—	—	—	—	—
	Δ RMR [%] R^2^	—	—	0.92 ***	—	—
	Δ BMR [%] R^2^	—	0.41 *	—	—	—
Somatic	Δ BM [%] R^2^	—	0.44 **	—	—	0.89 ***
	Δ FAT [%] R^2^	—	—	—	—	—
	Δ FFM [%] R^2^	—	—	—	—	0.88 ***
	Δ TBW [%] R^2^	—	0.19	—	—	—
	Δ BSA/BM [%] R^2^	—	—	—	—	—
Circulatory	Δ SBP [%] R^2^	—	—	—	—	—
	Δ DBP [%] R^2^	—	—	0.56 **	—	—

Note: Δ—changes; BM—body mass [%]; FAT—body fat [%]; FFM—fat-free mass [%]; SBP—systolic blood pressure [%]; DBP—diastolic blood pressure [%]; TBW—total body water [%]; C—whole-body thermal conductivity [%]; g—internal-to-skin temperature gradient [%]; Ti—internal temperature [%]; MTB—mean body temperature [%]; MTsk—mean skin temperature [%]; VO_2_—oxygen uptake [%]; BMR—basal metabolic rate [%]; RMR—resting metabolic rate [%]; R^2^—R^2^ predictors in regression analysis model, *—(*p* ≤ 0.05), **—(*p* ≤ 0.01), ***—(*p* ≤ 0.005).

## Data Availability

The data presented in this study are available upon request from the corresponding author.

## References

[1] Vinales K.L., Begaye B., Thearle M.S., Krakoff J., Piaggi P. (2019). Core body temperature, energy expenditure, and epinephrine during fasting, eucaloric feeding, and overfeeding in healthy adult men: Evidence for a ceiling effect for human thermogenic response to diet. Metab. Clin. Exp..

[2] Fullmer S., Benson-Davies S., Earthman C.P., Frankenfield D.C., Gradwell E., Lee P.S., Piemonte T., Trabulsi J. (2015). Evidence analysis library review of best practices for performing indirect calorimetry in healthy and non-critically ill individuals. J. Acad. Nutr. Diet..

[3] Kenny G.P., Journeay W.S. (2010). Human thermoregulation: Separating thermal and nonthermal effects on heat loss. Front. Biosci..

[4] Heymsfield S.B., Smith B., Dahle J., Kennedy S., Fearnbach N., Thomas D.M., Bosy-Westphal A., Müller M.J. (2021). Resting Energy Expenditure: From Cellular to Whole-Body Level, a Mechanistic Historical Perspective. Obesity.

[5] Benedict F.G. (1915). Factors Affecting Basal Metabolism. J. Biol. Chem..

[6] White C.R., Seymour R.S. (2005). Allometric scaling of mammalian metabolism. J. Exp. Biol..

[7] Taylor N.A.S., Notley S.R. (2018). Morphological and Physiological Considerations for the Modelling of Human Heat Loss. Theory and Applications of Heat Transfer in Humans.

[8] Rikke B.A., Johnson T.E. (2004). Lower body temperature as a potential mechanism of life extension in homeotherms. Exp. Gerontol..

[9] Song B., Thomas D.M. (2007). Dynamics of starvation in humans. J. Math. Biol..

[10] MacLean P.S., Bergouignan A., Cornier M.A., Jackman M.R. (2011). Biology’s response to dieting: The impetus for weight regain. Am. J. Physiol.-Regul. Integr. Comp. Physiol..

[11] Catenacci V.A., Pan Z., Ostendorf D., Brannon S., Gozansky W.S., Mattson M.P., Martin B., MacLean P.S., Melanson E.L., Donahoo W.T. (2016). A randomized pilot study comparing zero-calorie alternate-day fasting to daily caloric restriction in adults with obesity. Obesity.

[12] Rosenbaum M., Leibel R.L. (2010). Adaptive thermogenesis in humans. Int. J. Obes..

[13] Pokora I., Grucza R., Kaciuba-Uściłko H. (1999). Influence of a low-carbohydrate diet on thermoregulatory responses to prolonged exercise in men. J. Therm. Biol..

[14] Bachman J.L., Deitrick R.W., Hillman A.R. (2016). Exercising in the Fasted State Reduced 24-Hour Energy Intake in Active Male Adults. J. Nutr. Metab..

[15] Severinsen T., Munch I.C. (1999). Body core temperature during food restriction in rats. Acta Physiol. Scand..

[16] Finnell J.S., Saul B.C., Goldhamer A.C., Myers T.R. (2018). Is fasting safe? A chart review of adverse events during medically supervised, water-only fasting. BMC Complement. Altern. Med..

[17] Most J., Redman L.M. (2020). Impact of calorie restriction on energy metabolism in humans. Exp. Gerontol..

[18] Tang D., Tang Q., Huang W., Zhang Y., Tian Y., Fu X. (2023). Fasting: From Physiology to Pathology. Adv. Sci..

[19] Dai Z., Zhang H., Wu F., Chen Y., Yang C., Wang H., Sui X., Guo Y., Xin B., Guo Z. (2022). Effects of 10-Day Complete Fasting on Physiological Homeostasis, Nutrition and Health Markers in Male Adults. Nutrients.

[20] Blundell J.E., Caudwell P., Gibbons C., Hopkins M., Naslund E., King N., Finlayson G. (2012). Role of resting metabolic rate and energy expenditure in hunger and appetite control: A new formulation. Dis. Model. Mech..

[21] Hopkins M., Gibbons C., Blundell J. (2022). Fat-free mass and resting metabolic rate are determinants of energy intake: Implications for a theory of appetite control. Philos. Trans. R. Soc. B.

[22] Stubbs R.J., Hopkins M., Finlayson G.S., Duarte C., Gibbons C., Blundell J.E. (2018). Potential effects of fat mass and fat-free mass on energy intake in different states of energy balance. Eur. J. Clin. Nutr..

[23] Hopkins M., Finlayson G., Duarte C., Whybrow S., Ritz P., Horgan G.W., Blundell J.E., Stubbs R.J. (2016). Modelling the associations between fat-free mass, resting metabolic rate and energy intake in the context of total energy balance. Int. J. Obes..

[24] Abreu-Vieira G., Xiao C., Gavrilova O., Reitman M.L. (2015). Integration of body temperature into the analysis of energy expenditure in the mouse. Mol. Metab..

[25] Sohal R.S., Weindruch R. (1996). Oxidative stress, caloric restriction, and aging. Science.

[26] Rikke B.A., Yerg J.E., Battaglia M.E., Nagy T.R., Allison D.B., Johnson T.E. (2003). Strain variation in the response of body temperature to dietary restriction. Mech. Ageing Dev..

[27] Landsberg L. (2012). Core temperature: A forgotten variable in energy expenditure and obesity?. Obes. Rev..

[28] Anton S.D., Moehl K., Donahoo W.T., Marosi K., Lee S.A., Mainous A.G., Leeuwenburgh C., Mattson M.P. (2018). Flipping the Metabolic Switch: Understanding and Applying the Health Benefits of Fasting. Obesity.

[29] Goldhamer A., Lisle D., Parpia B., Anderson S.V., Campbell T.C. (2001). Medically supervised water-only fasting in the treatment of hypertension. J. Manip. Physiol. Ther..

[30] Chatamra K., Daniel P.M., Lam D.K.C. (1984). The Effects of Fasting on Core Temperature, Blood Glucose and Body and Organ Weights in Rats. Q. J. Exp. Physiol..

[31] Gordon C.J. (2012). Thermal physiology of laboratory mice: Defining thermoneutrality. J. Therm. Biol..

[32] Nagashima K., Tokizawa K., Marui S., Uchida Y. (2019). Circadian Body Temperature Rhythm and the Interaction with Energy State. Homeostasis—An Integrated Vision.

[33] Arens E., Zhang H. (2006). The skin’s role in human thermoregulation and comfort. Thermal and Moisture Transport in Fibrous Materials.

[34] Moran D.S., Pandolf K.B., Shapiro Y., Heled Y., Shani Y., Matthew W.T., Gonzales R.R. (2001). An environmental stress index (ESI) as a substitute for the wet bulb globe temperature (WBGT). J. Therm. Biol..

[35] Nishi Y. (1981). Measurement of thermal balance in man. Bioeng. Therm. Physiol. Comf..

[36] Du Bois D., Du Bois E.F. (1916). A formula to estimate the approximate surface area if height and weight be known. Arch. Intern. Med..

[37] Burton A. (1934). The application of the theory of heat flow to the study of energy metabolism. J. Nutr..

[38] Stolwijk J.A.J., Hardy J.D. (1966). Temperature regulation in man—A theoretical study. Pflüg. Arch..

[39] Lakens D. (2013). Calculating and reporting effect sizes to facilitate cumulative science: A practical primer for *t*-tests and ANOVAs. Front. Psychol..

[40] McIntosh R., Anderson V. (2010). A comprehensive tissue properties database provided for the thermal assessment of a human at rest. Biophys. Rev. Lett..

[41] Nakamura Y., Nakamura K. (2018). Central regulation of brown adipose tissue thermogenesis and energy homeostasis dependent on food availability. Pflug. Arch..

[42] Dulloo A.G., Jacquet J., Montani J.P., Schutz Y. (2012). Adaptive thermogenesis in human body weight regulation: More of a concept than a measurable entity?. Obes. Rev..

[43] Dulloo A.G., Schutz Y. (2015). Adaptive Thermogenesis in Resistance to Obesity Therapies: Issues in Quantifying Thrifty Energy Expenditure Phenotypes in Humans. Curr. Obes. Rep..

[44] Hall K.D. (2010). Predicting metabolic adaptation, body weight change, and energy intake in humans. Am. J. Physiol. Endocrinol. Metab..

[45] Letkiewicz S., Pilis K., Ślęzak A., Pilis A., Pilis W., Żychowska M., Langfort J. (2021). Eight days of water-only fasting promotes favorable changes in the functioning of the urogenital system of middle-aged healthy men. Nutrients.

[46] Dulloo A.G. (2021). Physiology of weight regain: Lessons from the classic Minnesota Starvation Experiment on human body composition regulation. Obes. Rev..

[47] Gallagher D., Albu J., He Q., Heshka S., Boxt L., Krasnow N., Elia M. (2006). Small organs with a high metabolic rate explain lower resting energy expenditure in African American than in white adults. Am. J. Clin. Nutr..

[48] Palmer B.F., Clegg D.J. (2021). Starvation Ketosis and the Kidney. Am. J. Nephrol..

[49] Dulloo A.G., Jacquet J. (1998). Adaptive reduction in basal metabolic rate in response to food deprivation in humans: A role for feedback signals from fat stores. Am. J. Clin. Nutr..

[50] Heshka S., Yang M.U., Wang J., Burt P., Pi-Sunyer F.X. (1990). Weight loss and change in resting metabolic rate. Am. J. Clin. Nutr..

[51] Rachakonda V.P., DeLany J.P., Kershaw E.E., Behari J. (2019). Impact of hepatic steatosis on resting metabolic rate and metabolic adaptation in response to intentional weight loss. Hepatol. Commun..

[52] Said Camilleri J., Farrugia L., Curto S., Rodrigues D.B., Farina L., Caruana Dingli G., Bonello J., Farhat I., Sammut C.V. (2022). Review of Thermal and Physiological Properties of Human Breast. Tissue Sens..

[53] Nahon K.J., Boon M.R., Doornink F., Jazet I.M., Rensen P.C.N., Abreu-Vieira G. (2017). Lower critical temperature and cold-induced thermogenesis of lean and overweight humans are inversely related to body mass and basal metabolic rate. J. Therm. Biol..

[54] Taylor N.A., Machado-Moreira C.A., van den Heuvel A.M., Caldwell J.N. (2014). Hands, and feet: Physiological insulators, radiators and evaporators. Eur. J. Appl. Physiol..

